# Huanglian-Hongqu herb pair improves nonalcoholic fatty liver disease via NF-κB/NLRP3 pathway in mice: network pharmacology, molecular docking and experimental validation

**DOI:** 10.1186/s41065-024-00316-0

**Published:** 2024-04-03

**Authors:** Zheng Wang, Hairong Qiu, Yang Yang, Yueyu Zhang, Taiguo Mou, Xiaobo Zhang, Yong Zhang

**Affiliations:** 1 College of Traditional Chinese Medicine and Health Service, Shanxi Datong University, Datong, China; 2 Department of Chinese Medicine, Medical School, Hubei Minzu University, Enshi, China; 3 Institute of Traditional Chinese Medicine, Sichuan Academy of Chinese Medicine Sciences, Chengdu, China; 4 College of Public health, Chengdu University of Traditional Chinese Medicine, Chengdu, China; 5 School of Basic Medicine, Chengdu University of Traditional Chinese Medicine, Chengdu, China; 6 Traditional Chinese Medicine department, Chinese Medicine Hospital of Chenghua, Chengdu, China

**Keywords:** Huanglian-Hongqu herb pair, Nonalcoholic fatty liver disease, Network pharmacology, Molecular docking, NF-κB/NLRP3 pathway

## Abstract

The Huanglian-Hongqu herb pair (HH) is a carefully crafted traditional Chinese herbal compound designed to address disorders related to glucose and lipid metabolism. Its primary application lies in treating hyperlipidemia and fatty liver conditions. This study explored the potential mechanism of HH in treating non-alcoholic fatty liver disease (NAFLD) through network pharmacology, molecular docking, and in vivo animal experiments. Ultrahigh performanceliquid chromatography-quadrupole/orbitrapmass spectrometry (UPLC-Q-TOF-MS) was employed to identify the chemical composition of HH. Network pharmacology was used to analyze the related signaling pathways affected by HH. Subsequently, the prediction was verified by animal experiment. Finally, we identified 29 components within HH. Network pharmacology unveiled interactions between HH and 153 NAFLD-related targets, highlighting HH’s potential to alleviate NAFLD through NF-κB signaling pathway. Molecular docking analyses illuminated the binding interactions between HH components and key regulatory proteins, including NF-κB, NLRP3, ASC, and Caspase-1. In vivo experiments demonstrated that HH alleviated NAFLD by reducing serum and liver lipid levels, improving liver function, and lowering inflammatory cytokine levels in the serum. Moreover, HH administration downregulated mRNA and protein levels of the NF-κB/NLRP3 pathway. In conclusion, our findings demonstrated that HH has potential therapeutic benefits in ameliorating NAFLD by targeting the NF-κB/NLRP3 pathway, facilitating the broader application of HH in the field of NAFLD.

## Introduction

Non-alcoholic fatty liver disease (NAFLD) is a liver injury caused by metabolic stress, characterized by excess triglyceride (TG) accumulation in liver cells [1]. On a global scale, NAFLD’s prevalence has surged to 20-30%, posing a serious public health issue [2, 3]. The progression of NAFLD can lead to the potential for severe consequences, including liver disease, disability, and mortality, and is robustly associated with type 2 diabetes mellitus (T2DM), atherosclerotic cardiovascular disease, and colorectal tumor [4]. The high prevalence of obesity and metabolic syndrome has transformed NAFLD into a major chronic liver disease [5]. Currently, no authorized medications are available for the treatment of NAFLD, necessitating the development of effective therapeutic strategies and pharmaceutical interventions [6].

Traditional Chinese medicine formulas (TCM) have been effectively used for decades to treat NAFLD, benefiting a large number of patients by ameliorating symptoms, reducing enzyme levels, and protecting the liver [7, 8]. Among these formulations, the Huanglian-Hongqu herb pair (HH) stands out as a noteworthy herbal combination in TCM, comprising Huanglian (*Coptis chinensis* Franch.) and Hongqu (red yeast rice). First introduced in the *Extensive Notes on Medicine from Xian Xing Studio* by Miao Xi-yong during the Ming Dynasty, HH has been primainly used for treating metabolic diseases such as hyperlipidemia, NAFLD, and obesity. Research by Tong et al. underscored the efficacy of the Huanglian-Hongqu combination in targeting insulin resistance, a central feature of fatty liver, and improving lipid deposition in the liver [9]. Similarly, Chen et al. observed that HH reduces blood lipid levels and restores normal liver functions in patients with hyperlipidemia complicated by abnormal liver function [10]. Moreover, contemporary pharmaceutical studies have proposed that Huanglian, a constituent of HH, exhibits blood lipid-regulating, anti-atherosclerosis, hypoglycemic, and antioxidant activities [11]. Berberine, a main ingredient of Huanglian, has been proven effective in improving NAFLD, regulating lipid metabolism, and alleviating liver injury [12, 13]. Hongqu also contibutes with its beneficial lipid-lowering effect, antioxidant and anti-diabetic activities, and ability to reduce insulin resistance [14]. Given the intricate nature of NAFLD’s underlying mechanisms, the primary focus of pharmacotherapy is to mitigate insulin resistance, coupled with a secondary objective of diminishing hepatic lipid accumulation [15]. In accordance with this rationale, our approach involves the concurrent administration of both drugs to address NAFLD. Despite the promising aspects of HH, its efficacy and mechanism for treating NAFLD have received less attention and remain insufficiently reported. Therefore, further research in this field is warranted.

Because the Chinese herbal formula (CHF) is complex, understanding how these herbs act on multiple targets and produce synergistic effects poses a considerable challenge [16]. Fortunately, network pharmacology, an emerging discipline, offers a potent tool to predict the mechanism of Chinese medicines, especially compound formulas [17]. In this study, we employed ultrahigh performanceliquid chromatography-quadrupole/orbitrapmass spectrometry (UPLC-Q-TOF-MS), network pharmacology, and animal experiment to uncover the efficacy and potential mechanism of HH in the treatment of NAFLD. Through this research, we aim to uncover the molecular mechanisms underlying HH’s therapeutic effect on NAFLD.

## Materials and methods

### Materials

Huanglian (Sichuan Guoqiang Chinese Medicine Granule Co., Ltd., Lot No.: 21,090,101) and Hongqu (Yaan Xunkang Pharmaceutical Co., Ltd., Lot No.: 210,501). HFD with 60 kcal % fat in total energy (D12492) was purchased from Chengdu Paishang Feike Biotechnology Co., Ltd. The following kits and antibodies were used in the study: total Cholesterol (TC) (No. 105-000448-00), TG (No. 105-000449-00), low-Density Lipoprotein Cholesterol (LDL-C) (No. 105-000464-00), high density lipoprotein cholesterol (HDL-C) (No. 105-000463-00), glutamic pyruvic transaminase (ALT) (No. 105-000442-00), and aspartate aminotransferase (AST) (No. 105-000443-00) were ordered from Mindray Biomedical Electronics Co., Ltd. (Shenzhen, China). NF-κB was purchased from CST (#8242), NLRP3 (bs-0169R), Caspase-1 (bs-0169R), and ASC (bs-6741R) were obtained from Bioss (Beijing, China), β-Actin (ab8226) and p-NF-κB (ab76302) was purchased from Abcam, Cleaved-Caspase-1 (AF4005) and Cleaved-IL-1β (AF4006) was purchased from Jiangsu Qinke Biological Research Center Co., Ltd (Wuhan, China). Chromatograph: UltiMate 3000 RS, Thermo Fisher Scientific (China) Co., Ltd. Mass spectrometer: Q Exactive high resolution mass spectrometer, Thermo Fisher Scientific (China) Co., Ltd. Chromatographic column: AQ-C18, 150 × 2.1 mm, 1.8 μm, Welch.

### Identifier of active ingredients and targets of HH

Hongqu (10 g) was finely powdered and mixed with Huanglian (10 g). The mixture was then decocted twice with a 10-fold volume of water for 30 min each time. After removing the leaves, the drug suspension was retained, concentrated, and freeze-dried, resulting in a HH yield of 16.25%. The lyophilized powder underwent analysis using UPLC-Q-TOF-MS under the following conditions: full mass at 70,000, dd-MS2 at 17,500, scan range from 150.0 to 2000.0 m/z, spray voltage at 3.8 kV (positive), and Capillary Temperature at 300$^ \circ {\rm{C}}$ [18]. The high-performance liquid chromatography (HPLC) analysis was refer to Su’s methord [19]. The SMILES data of the active ingredients were analyzed use Swiss TargetPrediction to identify the targets associated with HH.

### Acquisition of NAFLD-associated targets

NAFLD-associated targets were gathered from two databases: DisGeNET and GeneCards, utilizing “Non-alcoholic Fatty Liver Disease” as the search keyword. By intersecting NAFLD-associated targets with those related to HH, we successfully identified potential action targets of HH in treating NAFLD.

### Protein-protein interaction (PPI) analysis

The STRING database (https://cn.string-db.org/) is a resource for Protein-Protein Interactions (PPI) analysis. In the study, we imported the overlapping targets into the String database. To ensure reliability, a minimum combined score of 0.700 was applied, and the research species was limited to Homo Sapiens. Subsequently, PPI data files were obtained, exported, and then imported into Cytoscape 3.7.1 software for further analysis. To identify core target genes, network topology analysis was performed using the Cyto-hubba plug-in.

### Network construction

The active ingredients and their corresponding targets obtained were recorded in Excel files, and a “Drug-Ingredient-Target-Disease” network was constructed using Cytoscape 3.7.1 software. In this network, nodes were employed to signify the drug, active ingredients, and targets, while edges symbolized the interactions between these nodes.

### Gene ontology (GO) and kyoto encyclopedia of genes and genomes (KEGG) pathway enrichment analysis

The overlapping targets underwent GO and KEGG enrichment analyses utilizing the Metascape website (http://www.metascape.org), and the results were visualized using the Bioinformatics website (https://www.bioinformatics.com.cn/). From the analyses, the top 10 GO terms and the top 20 KEGG signaling pathways were selected for in-depth investigation.

### Molecular docking

To investigate the binding affinities and interaction modes between the active compounds and the candidate targets, we peformanced molecular docking. The protein’s molecular structures were obtained from the PubChem Compound database, while the 3D coordinates of NF-κB (PDB ID: 1LE9; resolution: 3.00Å), NLRP3 (PDB ID: 6NPY; resolution: 3.80Å), ASC (PDB ID: 6N1H; resolution: 3.17Å), and Caspase-1 (PDB ID: 2FQQ; resolution: 3.30Å) were obtained from the protein data bank (PDB) database. For the docking analysis, all protein and molecular files were converted into PDBQT format, excluding water molecules while adding polar hydrogen atoms. The molecular docking studies were performed using Autodock Vina 1.2.2, with binding energy serving as a metric to assess the binding activity between the ligand small molecules and the receptor protein. Generally, a binding energy of <-4.25 kcal/mol indicates a certain level of binding activity, while <-5.0 kcal/mol suggests a favorable binding activity between the ligands and the respective proteins.

### Animal experiment

Male C57BL/6 mice, 6 weeks old and provided by SPF (Beijing) Biotechnology Co., Ltd., were utilized for the study. The mice were housed in a specific-pathogen-free (SPF) animal facility under controlled conditions: a temperature of (22 ± 1) $^ \circ {\rm{C}}$, a relative humidity of 55-60%, environmental noise maintained below 60 dB, and a consistent 12-hour light/12-hour dark cycle. The study was carried out in accordance with the NC3Rs ARRIVE Guidelines. The animal experiment was thoroughly reviewed and approved by the Animal Experimentation Ethics Committee of Chengdu University of Traditional Chinese Medicine on June 13, 2023 (Ethics No. 2022-11).

### HH preparation and NAFLD modeling

After a 7-day period of adaptive feeding, the mice were randomly allocated into two main groups: the control group (*n* = 12) and the model group (*n* = 40). The control group received a standard diet, whereas the model group was fed a high-fat diet. After a 16-week feeding period, we assess the success of the model. Any mice that did not meet the specific criteria were excluded from the subsequent analysis. The remaining mice were then randomly divided into five treatment groups: control group (*n* = 6), NAFLD group (*n* = 6), low-dose HH group (HH-L, 250 mg/kg, *n* = 6), middle-dose HH group (HH-M, 500 mg/kg, *n* = 6) - this dose was selected based on normal human dose levels, and high-dose HH group (HH-H, 1000 mg/kg, *n* = 6). The mice in the HH-L, HH-M, and HH-H groups received their respective treatment drugs orally for a duration of 6 weeks. At the conclusion of the 6-week administration period, the mice were humanely euthanized using 10% pentobarbital sodium.

### Serum levels of TC, TG, LDL-C, HDL-C, ALT, and AST

A Mindray automatic biochemical analyzer was utilized to measure the biochemical indices in the tail vein blood of mice after a 12-hour fast, including serum lipid level (TC, TG, LDL-C, HDL-C) and liver function (ALT, AST).

### Liver levels of TC, TG, and free fatty acid (FFA)

For the analysis of liver tissues, a full homogenization was carried out using 9-fold ice-cold normal saline under freezing conditions. The homogenized samples were then centrifuged at 3,000 r/min and 4 $^ \circ {\rm{C}}$ for 20 min. The protein content was measured using the BCA method. The levels of TC, TG, and FFA were analyzed following the manufacturer’s instructions.

### Histopathology

The liver tissues was fixed in 4% paraformaldehyde and underwent dehydration, paraffin embedding, sectioning, and treatment with hematoxylin and eosin (HE) staining. The liver pathomorphology was examined using a light microscope. Additionally, another part of the liver tissues was immersed in a 30% sucrose solution for 48 h, followed by dehydration, optimal cutting temperature compound (OTC) embedding, and sectioning into 5-µm sections. These sections were then washed with 60% isopropanol and subsequently stained with the Oil-Red-O staining solution as per the manufacturer’s instructions.

### ELISA for serum IL-6 and TNF-α

The serum inflammatory factors, interleukin-6 (IL-6), and tumor necrosis factor-alpha (TNF-α) were quantified using an enzyme-linked immunosorbent assay (ELISA) kit (Multi Sciences, Hangzhou, China) following the manufacturer’s instructions.

### qPCR analysis

For RNA analysis, appropriate cryopreserved liver tissues were utilized to extract total RNA, which was subsequently subjected to reverse transcription to synthesize cDNA for further qPCR amplification. The primer sequences essential for qPCR were custom-designed and synthesized by Sangon Biotech (Shanghai, China). The PCR conditions were set as follows: an initial denaturation step at 95 $^ \circ {\rm{C}}$ for 5 min, followed by 40 cycles of denaturation at 95 $^ \circ {\rm{C}}$ for 10 s, annealing at 60 $^ \circ {\rm{C}}$ for 30 s, and extension from 65 $^ \circ {\rm{C}}$ to 95 $^ \circ {\rm{C}}$ with a 0.5 $^ \circ {\rm{C}}$/5-second gradient. The relative mRNA expression was calculated using the 2^−△△CT^ method. The primer sequences are detailed in Table 1.

**Table 1:** Primer sequences

Target genes	Primer sequence (5’-3’)
IL-1β	FORWARD: CCCTTGACTTGGGCTGT
	REVERSE: CGAGATGCTGCTGTGAGA
IL-18	FORWARD: AACGAATCCCAGACCAGAC
	REVERSE: AGAGGGTAGACATCCTTCCAT
IL-6	FORWARD: CTCCCAACAGACCTGTCTATAC
	REVERSE: CCATTGCACAACTCTTTTCTCA
β-Actin	FORWARD: CTGTGTGGATTGGTGGCTCT
	REVERSE: CAGCTCAGTAACAGTCCGCC

### Western blot analysis

Cryopreserved liver tissues (100 mg) were milled on ice and lysed to obtain total protein lysates.The protein quantification was performed using the bicinchoninic acid assay (BCA) protein assay kit. Proteins of varying molecular weights were separated using sodium dodecyl sulfate polyacrylamide gel electrophoresis (SDS-PAGE) and transferred to a polyvinylidene fluoride (PVDF) membrane. Following 1 h of blocking in 5% skim milk at 37 $^ \circ {\rm{C}}$, the membrane was sequentially incubated with specific primary antibodies (4 $^ \circ {\rm{C}}$, overnight) and secondary (37 $^ \circ {\rm{C}}$, 2 h) antibodies. TBST washing was performed before the addition of secondary antibodies. Protein bands were detected using chemiluminescence, and the optical density (OD) was calculated using the Image J software. Primary antibodies used included NF-κB (1:1000), p-NF-κB (1:1000), NLRP3 (1:1000), ASC (1:1000), and Caspase-1 (1:1000), with β-Actin used as the internal reference. The experiment was repeated three times.

### Data and statistical analysis

The results are presented as mean ± SEM (standard error of the mean). For statistical analysis of multiple comparisons, one-way analysis of variance (ANOVA) with the Dunnett multiple comparisons test was used, and statistical significance was considered at *p* < 0.05. All statistical analyses were performed using GraphPad Prism.

## Results

### Targets of HH for the treatment of NAFLD

**Fig. 1:**
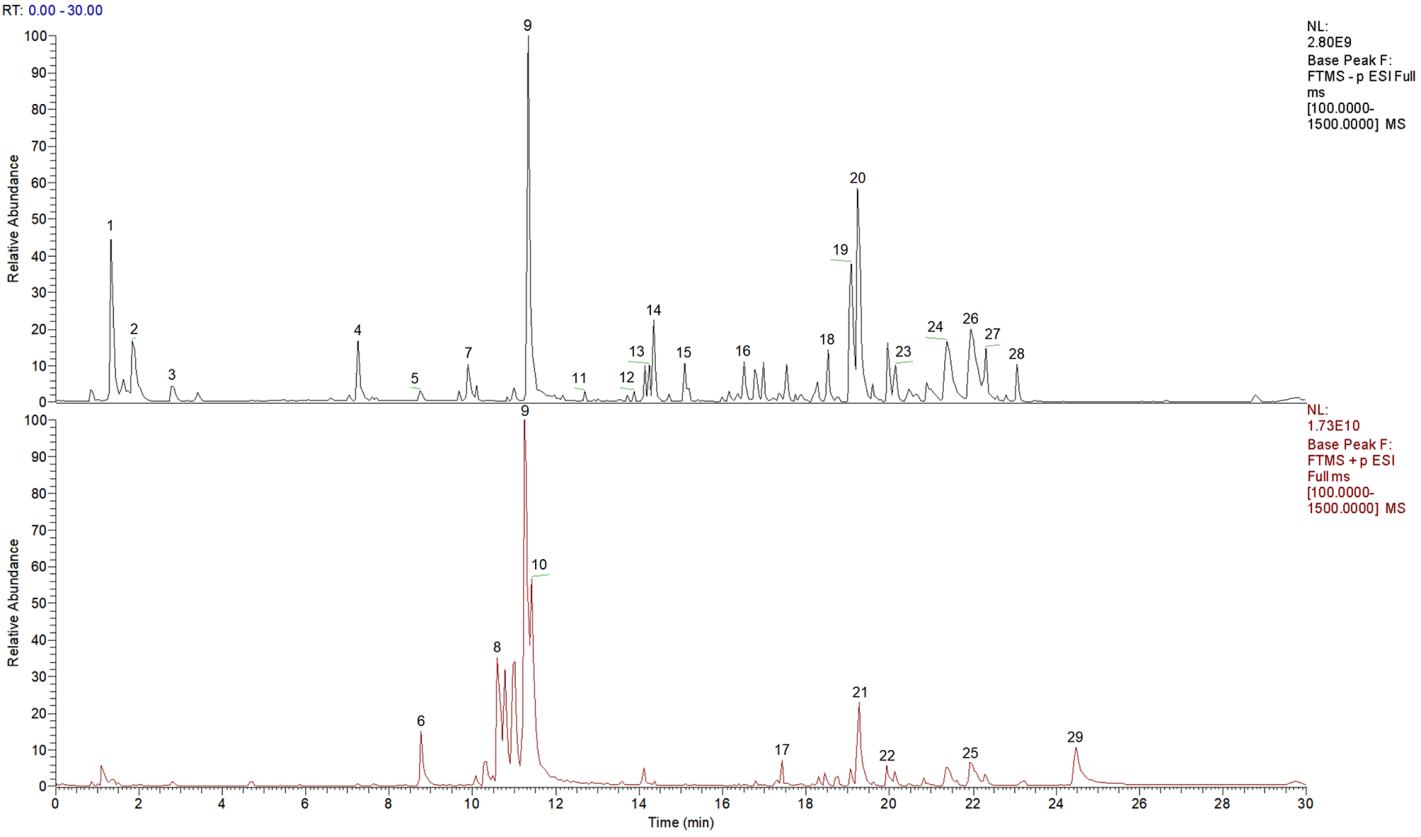
The total ion current diagram of HH

**Fig. 2:**
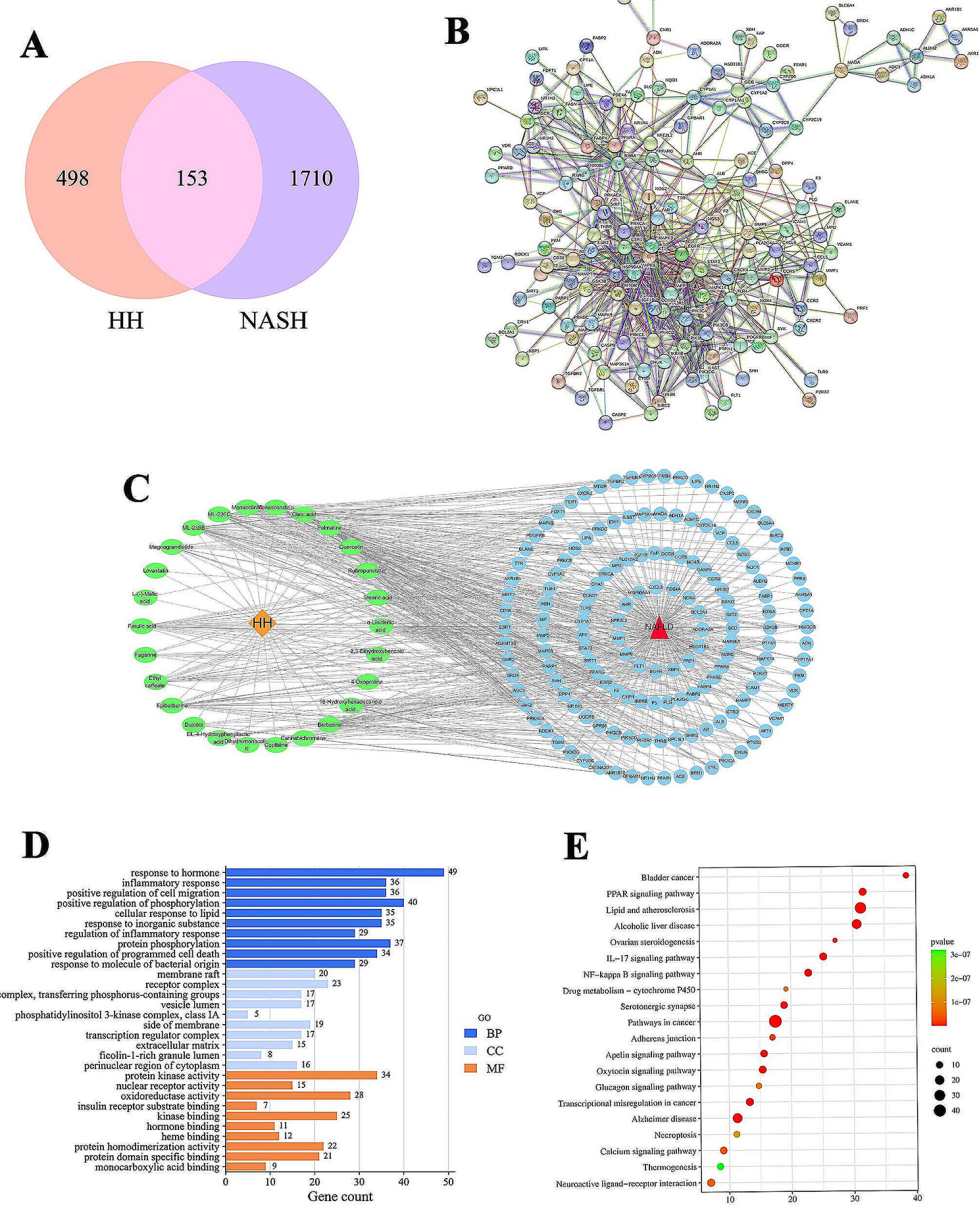
Network pharmacology analysis of HH in ameliorating NAFLD. (**A**) The Venn diagram. (**B**) PPI network. (**C**) Drug-component-target-disease network of HH. The orange rhombus node represents HH, the green oval nodes represent ingredients, the blue round nodes represent targets, and the red triangle node represents NAFLD. (**D**) GO analysis diagram. (**E**) KEGG pathway enrichment analysis diagram

In this study, we identified 29 chemical constituents ussing UPLC-Q-TOF-MS (Fig. 1; Table 2). The SMILES data of these compounds were then analyzed with Swiss TargetPrediction, resulting in the identification of 651 potential therapeutic targets after eliminating duplicates. To specifically focus on targets associated with NAFLD, we retrieved 434 targets from the DisGeNET database and an additional 1642 targets from the GeneCards database. By overlapping these datasets, we found 153 common targets shared by both sources. These shared targets suggest that HH may exert its effects on NAFLD by modulating these specific targets (Fig. 2A). To better understand the interactions between drug compounds and disease-associated targets, we constructed and visualized the network using Cytoscape 3.7.1 software (Fig. 2C). The result provided a comprehensive view of the targets through which HH may regulate NAFLD.

**Table 2:** Chemical compounds of HH identified by UPLC-Q-TOF-MS

No.	Compound name	Formula	Expected mass m/z	observed	DeltaMass [ppm]	RT(min)	Adducts
1	Dulcitol	C_6_H_14_O_6_	182.0790	182.0781	1.2	1.34	-
2	L-(-)-Malic acid	C_4_H_6_O_5_	134.0202	134.0203	0	1.84	-
3	4-Oxoproline	C_5_H_7_NO_3_	129.0413	129.0426	-0.8	2.78	-
4	2,3-Dihydroxybenzoic acid	C_7_H_6_O_4_	154.0266	154.0256	0.8	7.24	-
5	DL-4-Hydroxyphenyllactic acid	C_9_H_10_O_4_	182.0579	182.0572	4.2	8.74	-
6	2,3,4,9-Tetrahydro-1 H-β-carboline-3- carboxylic acid	C_12_H_12_N_2_O_2_	216.0899	216.0899	-0.1	8.76	+
7	Ferulic acid	C_10_H_10_O_4_	194.0580	194.0572	3.8	9.89	-
8	Coptisine	C_19_H_14_NO_4_	320.1260	320.1261	-0.4	10.59	+
9	Berberine	C_20_H_17_NO_4_	335.1158	335.1152	1.8	11.24	+, -
10	Epiberberine	C_20_H_18_ClNO_4_	372.1474	372.1436	-1.1	11.41	+
11	Magnograndiolide	C_15_H_22_O_4_	266.1552	266.1521	0.3	12.70	-
12	ML-236 C	C_18_H_26_O_3_	290.0790	290.0793	-0.8	13.87	-
13	Rubropunctatin	C_21_H_22_O_5_	354.2043	354.20433	-0.3	14.23	-
14	Monascorubrin	C_16_H_33_NO	255.2562	255.2562	0.1	14.34	-
15	Ethyl caffeate	C_11_H_12_O_4_	208.0732	208.0730	-0.9	15.08	-
16	Fagarine	C_13_H_11_NO_3_	230.1518	230.1515	1.2	16.51	-
17	Dihydromonacolin K	C_24_H_38_O_5_	406.1475	406.1476	-0.3	17.41	+
18	ML-236B	C_23_H_34_O_5_	390.2056	390.2016	0.8	18.52	-
19	Palmatine	C_21_H_22_NO_4_	352.0841	352.0795	3.2	19.09	-
20	Lovastatin	C_24_H_36_O_5_	404.2534	404.2563	0.3	19.24	-
21	Quercetin	C_15_H_10_O_7_	302.1882	302.1877	1.7	19.26	+
22	α-Linolenic acid	C_18_ H_30_ O_2_	278.2246	278.2245	0.3	19.93	+
23	Magnograndiolide	C_15_H_22_O_4_	266.1552	266.1551	0.3	20.15	-
24	Monacolin Q	C_19_H_22_O_2_	282.2559	282.2557	0.8	21.37	-
25	Cannabichromene	C_21_H_30_O_2_	314.2246	314.2245	0	21.93	+
26	Oleic acid	C_18_H_34_O_2_	282.2559	282.2557	0.8	21.95	-
27	N1-(3-Piperidinopropyl)-2-[(4-nitrobenzoyl)amino]benzamide	C_22_H_26_N_4_O_4_	410.1954	410.1980	-6.2	22.31	-
28	Trans-Petroselinic acid	C_18_H_34_O_2_	282.2559	282.2557	0.8	23.06	-
29	Stearic acid	C_18_H_36_O_2_	284.2715	274.2717	-0.7	24.48	+

### PPI network

In this phase of the study, we took the 153 overlap target genes and conducted PPI analysis by importing them into the STRING database. In the resulting network, nodes with deeper colors indicated higher degree values, signifying a greater number of interactions with other nodes. The network comprised 146 nodes and 651 edges, reflecting the relationships between different target genes. From this analysis, we identified the top 10 genes with the highest degree values, including AKT1, HSP90AA1, STAT3, EGFR, MAPK3, TLR4, PIK3R1, RXRA, ESR1, and ALB (Fig. 2B).

### Potential biological functions and pathways of HH for the treatment of NAFLD

To gain depper insight into the underlying mechanism of HH against NAFLD, we subjected the 153 common targets to GO annotation and KEGG pathway analysis. This comprehensive analysis aimed to elucidate the biological processesand specific pathways involved in HH’s therapeutic action on NAFLD. The GO annotation analysis revealed several highly enriched terms, such as inflammatory response, regulation of inflammatory response, protein phosphorylation, and cellular response to lipid, among others (Fig. 2D). Furthermore, the KEGG pathway analysis highlighted the top enriched pathways associated with NAFLD and the modulation of its targets by HH. These pathways included the PPAR signaling pathway, lipid and atherosclerosis, IL-17 signaling pathway, and NF-κB signaling pathway (Fig. 2E).

### Molecular docking evaluation

**Fig. 3:**
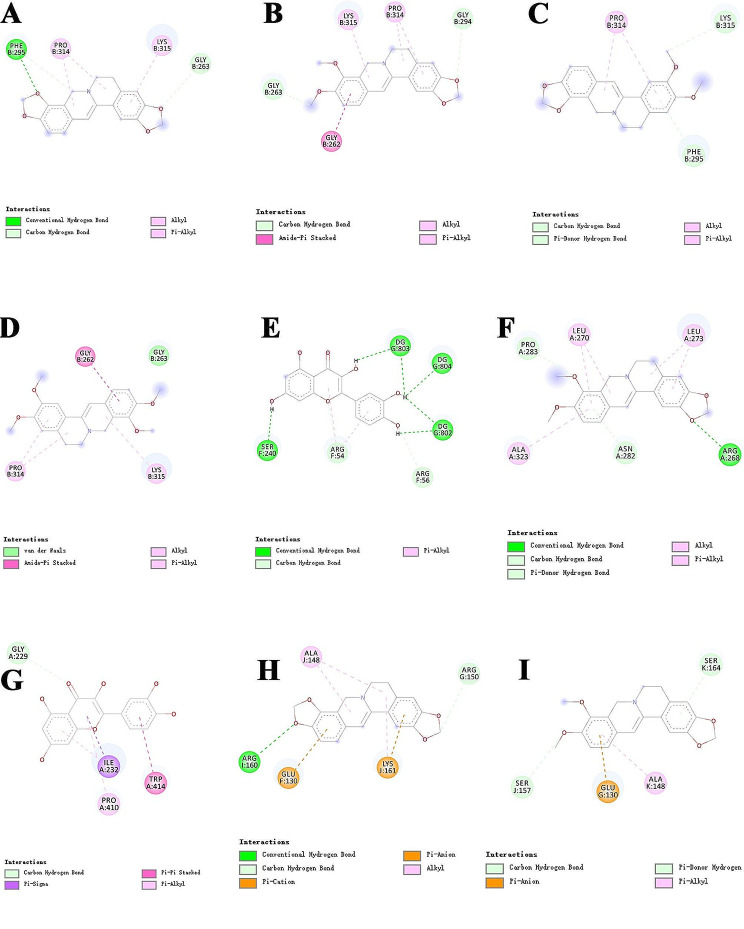
Binding mode of nine high-peak compounds with NF-κB, NLRP3, ASC, and Caspase-1. (**A**) Coptisine with NF-κB. (**B**) Berberine with NF-κB. (**C**) Epiberberine with NF-κB. (**D**) Palmatine with NF-κB. (**E**) Quercetin with NF-κB. (**F**) Berberine with NLRP3. (**G**) Quercetin with NLRP3. (**H**) Coptisine with ASC. (**I**) Berberine with ASC

To assess the candidate compounds’ affinity for their respective targets, a comprehensive molecular docking analysis was conducted. This process involved the use of Autodock Vina v.1.2.2 to determine the binding poses and interactions of seven high-peak compounds with four proteins. The data obtained from the molecular docking analysis were compiled in Fig. 3; Table 3. The results revealed that most of the compounds exhibited favorable binding energy, and the compound molecules formed conventional hydrogen bonds, pi-alkyl interactions, carbon-hydrogen bonds, and other intermolecular forces, indicating that HH could potentially regulate the key tergets of the NF-κB/NLRP3 signaling pathway.

**Table 3:** Molecular docking score of seven high-peak compounds of HH with NF-κB, NLRP3, ASC, and Caspase-1

Compounds	Binding Energy (kcal/mol)			
	NF-κB NLRP3 ASC Caspase-1			
Dulcitol	-5.50	-4.80	-4.40	-3.0
Coptisine	-8.90	-7.10	-8.50	-6.0
Berberine	-9.70	-7.80	-7.50	-5.20
Epiberberine	-8.60	-6.90	-7.10	-5.40
Palmatine	-8.70	-6.70	-6.80	-4.90
Lovastatin	-7.10	-5.70	-6.70	-5.10
Quercetin	-8.80	-7.40	-7.30	-5.40

### HH prevents the HFD-induced steatosis and liver injury in mice

**Fig. 4:**
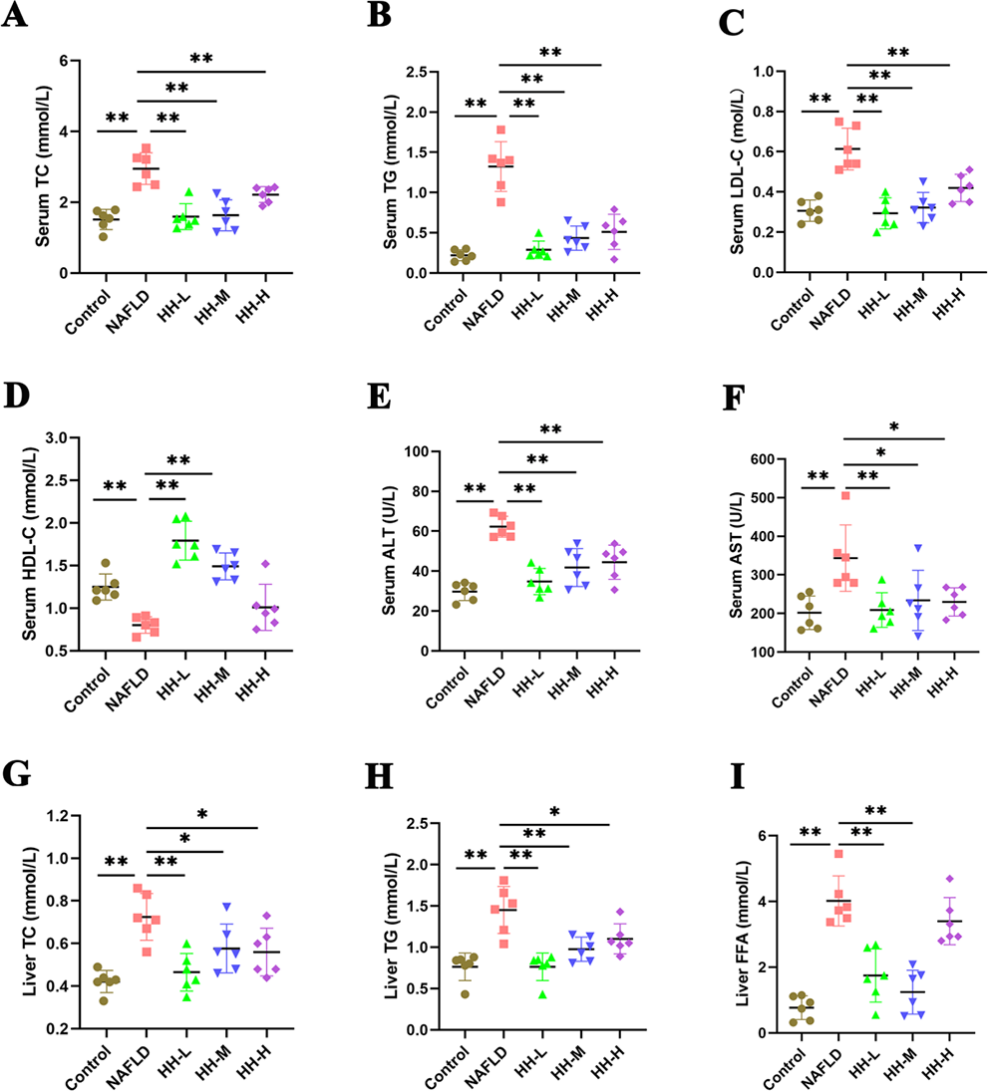
The effect of HH on serum lipid levels, liver function, and liver lipid in mice. The serum levels of TC (**A**), TG (**B**), LDL-C (**C**), HDL-C (**D**), liver function, ALT (**E**), AST (**F**), and liver lipid, TC (**G**), TG (**H**), FFA (**I**) in the mouse. *n* = 6 per group (Compared with NAFLD group: * *p* < 0.05, ** *p* < 0.01)

**Fig. 5:**
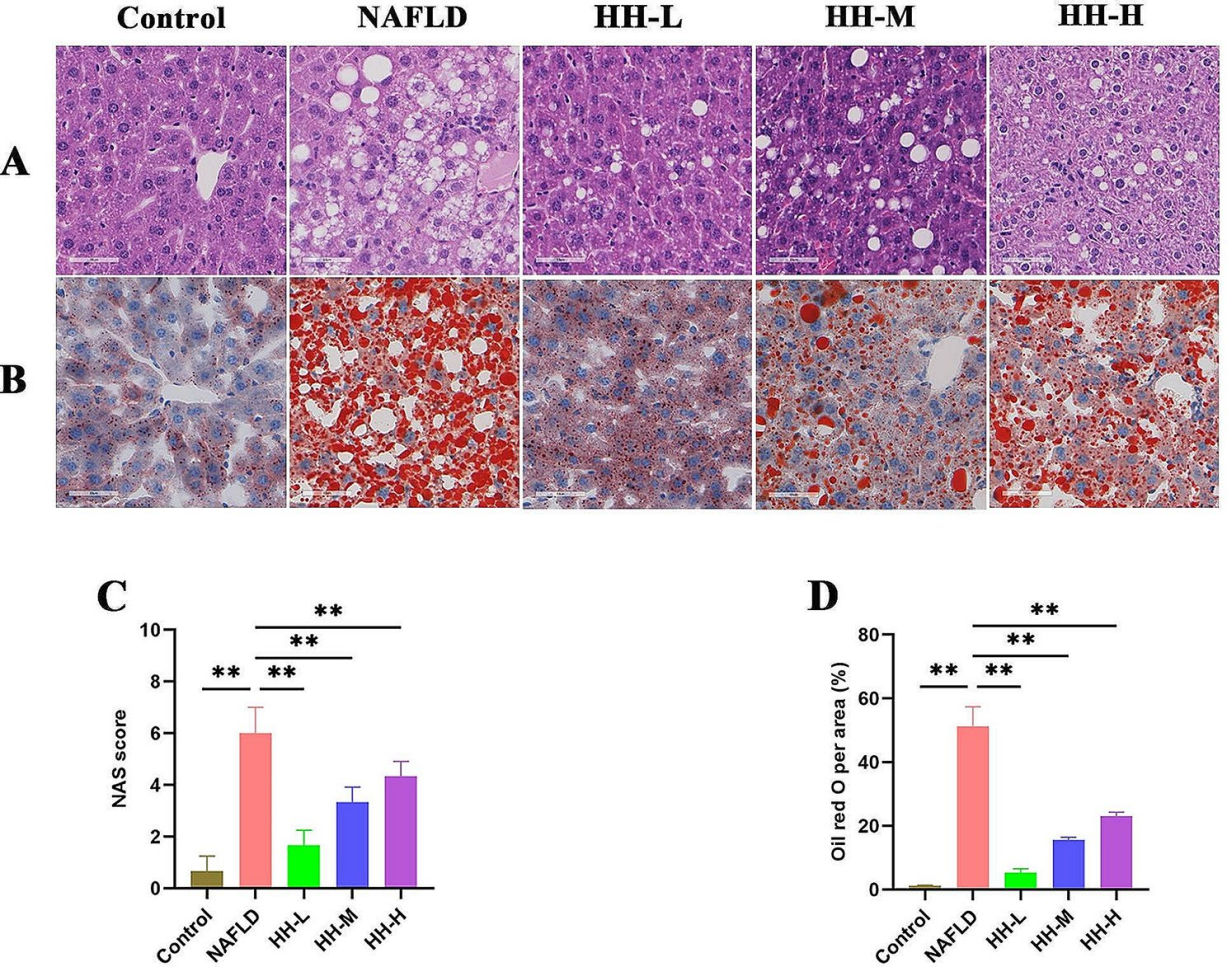
HH reduced liver lipid deposition (original magnification × 400). (**A**) HE staining. (**B**) Oil-Red-O staining. (**C**) NAS score. (**D**) Oil-Red-O per area

The HFD-fed mice exhibited increased blood lipid levels of TC, TG and LDL-C, while their HDL-C levels decreased. Additionally, the mice showed elevated liver indices (ALT, AST) and heightened liver lipid levels (TC, TG, FFA). However, after the oral administration of HH, there was a significant improvement in all these indices (Fig. 4A-I), Notably, in the low-dose group, the improvement in serum TG, LDL-C, as well as liver enzymes ALT and AST, was more pronounced compared to the medium and high-dose groups. Furthermore, Oil-Red-O and HE staining of liver sections revealed steatosis with hepatocyte swelling in the livers of mice in the NAFLD group. Remarkably, HH administration led to an improvement in the hepatic steatosis and hepatocyte swelling (Fig. 5A-D). These results suggest that HH could protect mice against HFD-induced NAFLD, with the low dosage possibly having a more favorable effect.

### HH reduces serum levels of inflammatory cytokines in mice

**Fig. 6:**
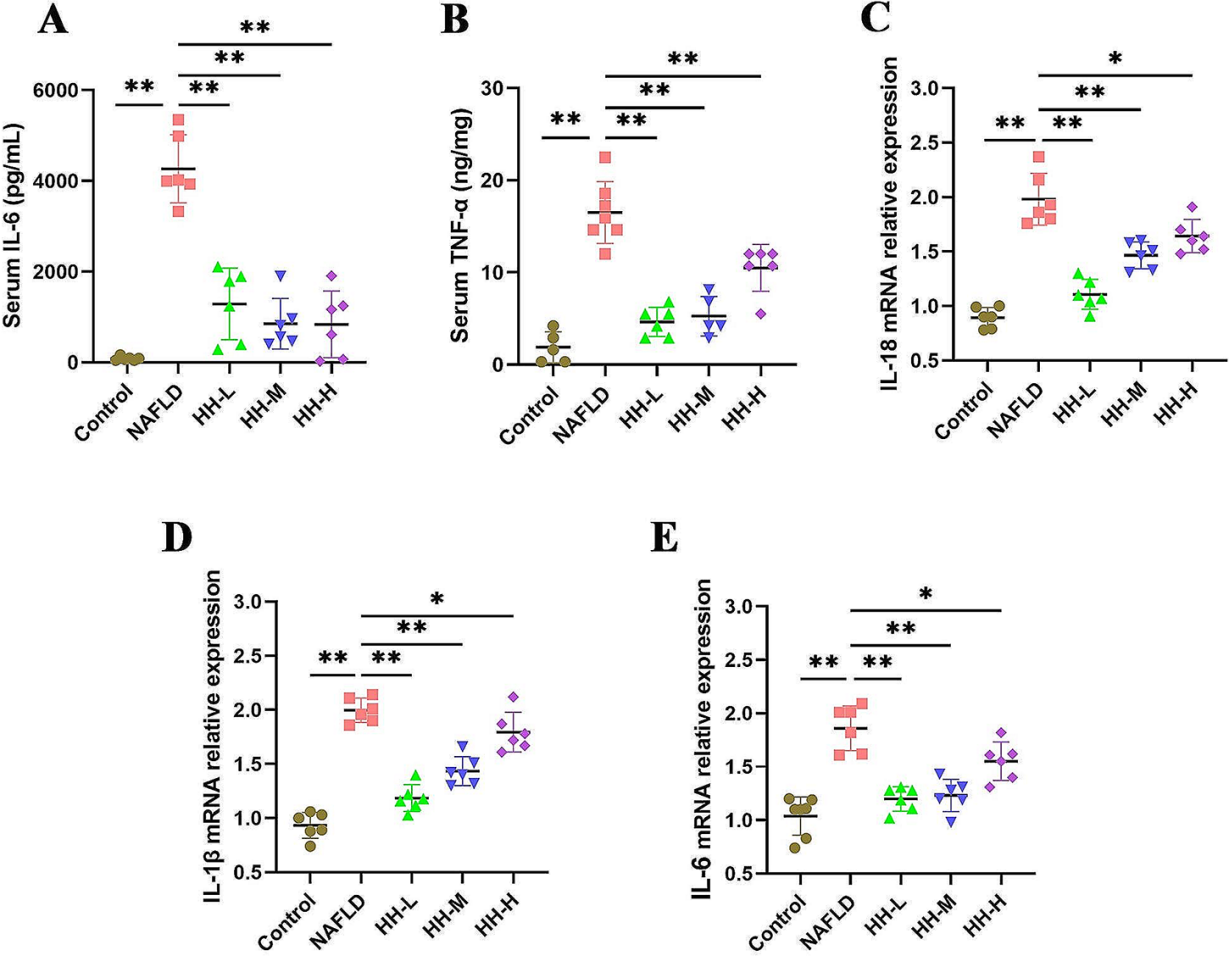
Serum inflammatory cytokine levels and liver relative mRNA expression of inflammatory cytokine. serum IL-6 level (**A**), serum TNF-α level (**B**), IL-18 relative mRNA expression (**C**), IL-1β relative mRNA expression (**D**), IL-6 relative mRNA expression (**E**)

Inflammation is recognized as a key factor driving the development of NAFLD and promoting liver injury. We measured the serum inflammatory cytokine levels, IL-6 and TNF-α in the NAFLD model and the control group using ELISA. The results demonstrated that the NAFLD mice had significantly higher levels of IL-6 and TNF-α compared to the Control group. Importantly, after HH administration, the serum levels (IL-6 and TNF-α) showed a noticeable decrease. The trend of TNF-α reduction in the HH-L group was superior to that in HH-M and HH-H groups. However, there seemed to be no significant difference in the improvement of serum IL-6 among the three groups (Fig. 6A-B).

### HH inhibiting the NF-κB/NLRP3 signaling pathway to alleviate liver inflammation

**Fig. 7:**
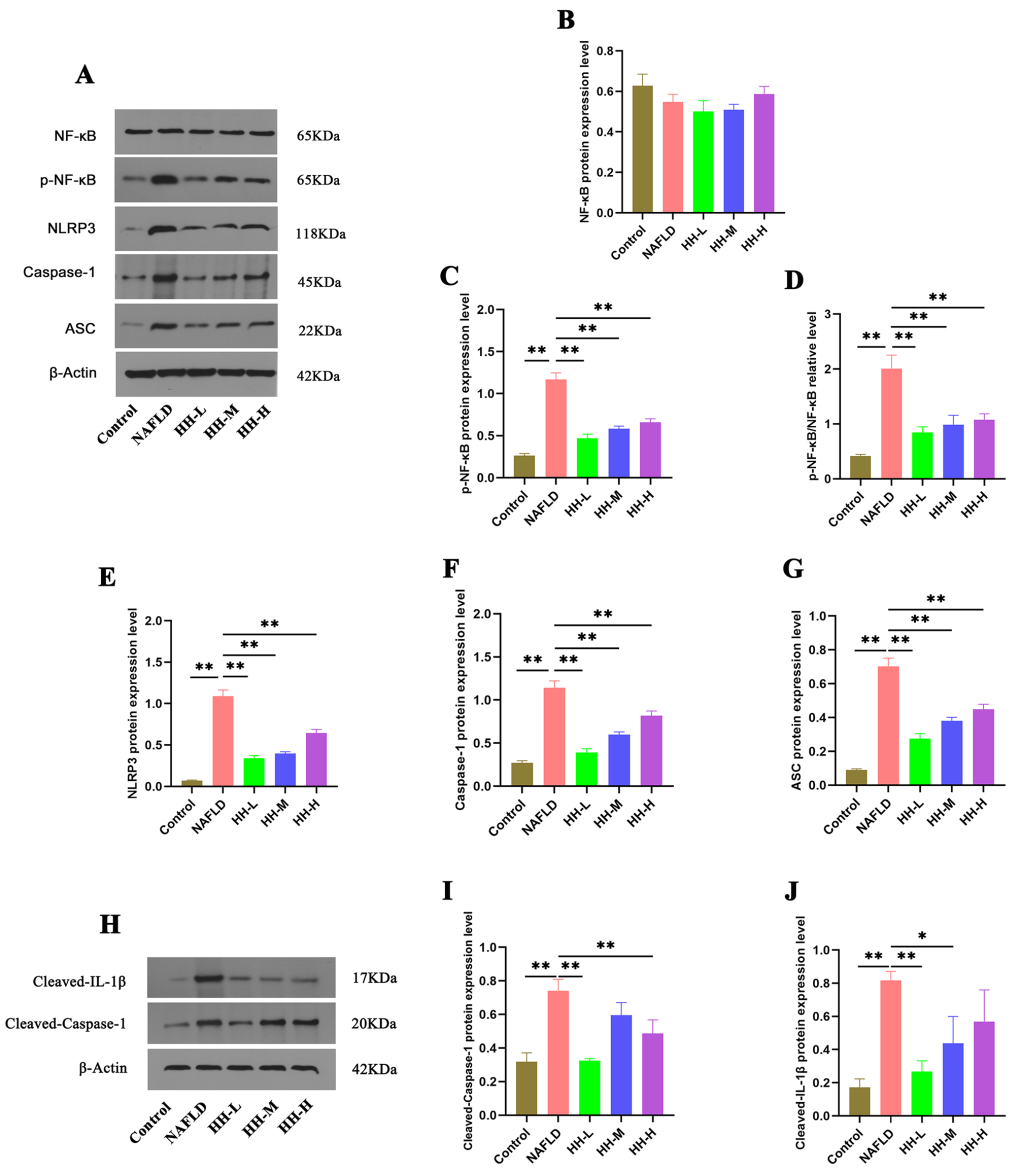
The protein expression levels of NF-κB/NLRP3 pathway analyzed by western blotting, *n* = 3 per group. (A-G) The expression levels of NF-κB, p-NF-κB, NLRP3, Caspase-1, and ASC. (H-J) The expression levels of Cleaved-Caspase-1 and Cleaved-IL-1β

Given that NLRP3 is a downstream target of NF-κB and the NF-κB/NLRP3 signaling pathway plays a pivotal role in NAFLD-related inflammation, we hypothesize that HH may exert potent anti-inflammatory effects by regulating this signaling pathway. To investigate this, we measured the mRNA levels of IL-18, IL-1β, and IL-6 in HFD-fed mice. As anticipated, the HFD-induced mice exhibited increased mRNA levels of these inflammatory cytokines. However, following HH intervention, there was a notable reversal in these levels, regardless of the HH dose administered (Fig. 6C-E). Furthermore, we assessed the protein levels of key components in the NF-κB/NLRP3 signaling pathway, including p-NF-κB, NLRP3, ASC, Caspase-1, Cleaved-Caspase-1, and Cleaved-IL-1β. In NAFLD mice, these protein levels were elevated. However, after HH administration, these levels were significantly decreased (Fig. 7A-J)

## Discussion

NAFLD has become the most prevalent chronic liver disease globally, posing a substantial burden on the public health system [20]. Its prevalence is further escalating due to the rising incidence of obesity, type 2 diabetes mellitus, and metabolic syndrome. Unfortunately, there are currently no FDA-approved drugs to address this medical condition. The pathogenesis of NAFLD is multifaceted, and drugs targeting a single pathway have exhibited limited efficacy [21, 22]. In light of these challenges, Chinese herbs have gained increasing attention for their potential effectiveness and minimal side effects [23]. Among them, the Chinese herbal compound HH has shown promise in NAFLD treatment; however, the precise mechanism remains poorly understood [24].

Chronic inflammation is a pivotal driving factor in the pathogenesis of NAFLD, characterized by abnormal production of proinflammatory cytokines and activation of inflammatory signaling pathways [25, 26]. NF-κB, a widely distributed transcription factor present in various cells, is crucial to the body’s inflammatory response [27]. Studies have shown that suppressing NF-κB can ameliorate inflammation in NAFLD [28]. The liver’s generation of reactive oxygen species (ROS) triggers the activation of the NF-κB complex, leading to IkB kinase-dependent IkB phosphorylation, which releases downstream inflammatory cytokine like TNF-α, culminating in inflammation [29]. NLRP3, a key palyer in NAFLD development, has received considerable attention in liver diseases. Animal experiments have shouwn upregulation of NLRP3, ASC, and Caspase-1 gene levels in the liver of NAFLD animals with steatosis. NLRP3 is highly activated and expressed during the progression from NAFLD to non-alcoholic steatohepatitis [30]. The NLRP3 inflammasome is typically activated via two signaling pathways. Exogenous deoxyadenosine monophosphates (DAMPs) bind with toll-like receptor 4 (TLR4), a member of the Toll-like receptors (TLR) family, promoting the phosphorylation of NF-κB inhibitor IκB [31]. Consequently, p65 and p50 dissociate from the nucleus, increasing NF-κB transcription and activating NLRP3, pro-IL-1β, and pro-IL-18 [32]. Alterations in ions (K^+^, Na^+^, Ca^2+^, Cl^−^), ROS production, and lysosome damage lead to the recruitment of NLRP3, promoting the conversion of pro-Caspase-1 to active Caspase-1, which then cleaves pro-IL-1β and pro-IL-18 to form mature IL-1β and IL-18 [33]. ASC acts as an adaptor protein, facilitating the recruitment of pro-caspase-1 to NLRP3. Caspase-1, a cysteine aspartic protease present in mammalian cells, plays a crucial role in initiating apoptosis in many cells and processing the precursors IL-1β and IL-18. Studies have demonstrated that knocking down Caspase-1 can protect against hepatocyte pyroptosis in NASH mice [34]. In NAFLD progression, excessive lipid accumulation triggers the generation of ROS, activating NF-κB and MAPK signaling pathway. Activated NF-κB translocates to the nucleus, modulating the expression of NLRP3, IL-1β, and other inflammatory and fibrotic cytokines [35]. NLRP3 binding to ASC activates Caspase-1, leading to the maturation of IL-1β. The upregulation of IL-1β exacerbates the inflammatory response, further promoting the development of NAFLD [36].

In this study, we investigated the potential mechanism by which HH treats NAFLD. A total of 651 treatment targets of HH were obtained, among which 153 were also associated with NAFLD. The functional analysis of these 153 overlapping targets revealed several enriched GO terms, including inflammatory response, regulation of inflammatory response, protein phosphorylation, and cellular response to lipid. Additionally, the top enriched KEGG pathways associated with NAFLD included PPAR signaling patway, lipid and atherosclerosis, IL-17 signaling patway, NF-κB signaling pathway, among others. Due to the crucial role of inflammation in NAFLD progression, we specifically focused our attention on the NF-κB signaling pathway. Additionally, given that the NLRP3 inflammasome acts as a downstream target of NF-κB, we further investigated the NF-κB/NLRP3 signaling pathway in subsequent in vivo experiments.

In animal experiments, HH demonstrated significant improvements in NAFLD, as evidenced by improved liver function, reduced serum lipid levels, and diminished liver lipid levels. These improvements resulted in a reduction in steatosis and inflammation in liver tissues, indicating HH’s therapeutic potential against NAFLD. Further investigations focused on the NF-κB/NLRP3 signaling pathway, which plays a crucial role in NAFLD. NAFLD mice exhibited elevated levels of p-NF-κB, NLRP3, ASC, Caspase-1, Cleaved-Caspase-1, and Cleaved-IL-1β in liver tissues, all of which were downregulated upon HH treatment. Interestingly, the low-dose group showed better results than the high-dose group, which did not display a clear dose-dependent pattern. In animal studies, medicinal herbs may not always exhibit dose-dependent effects. Furthermore, the specific doses of Huanglian and Hongqu in the high-dose group might have exceeded the maximum effective therapeutic dose, leading to an absence of correlation between dose increase and efficacy [37]. Additionally, low doses of Huanglian could promote gastrointestinal peristalsis and reduce insulin resistance, while high doses might cause abdominal distension [38, 39]. In the case of Hongqu, a large dose may increase appetite but result in inferior therapeutic effects compared to the low-dose group [40]. Moreover, the multi-component and multi-target characteristics of CHF are essential factors influencing drug efficacy. These complex interactions may lead to variations in the therapeutic direction at different doses and combinations of herbal components.

## Conclusion

The present study utilized UPLC-Q-TOF-MS, network pharmacology, and in vivo animal experiments to explore the effectiveness and molecular mechanism of HH in treating NAFLD. Our findings suggest that HH exertes its therapeutic effect against NAFLD through multiple targets, with the NF-κB/NLRP3 signaling pathway potentially playing a crucial role. These results contribute to a deeper understanding of HH’s mechanism of action in NAFLD treatment.

## Data Availability

Materials and data pertinent to this study are available upon request via email to the corresponding author.
